# *In Utero* Exposure to Dioxins and Dioxin-like Compounds and Anogenital Distance in Newborns and Infants

**DOI:** 10.1289/ehp.1205221

**Published:** 2012-11-19

**Authors:** Marina Vafeiadi, Silvia Agramunt, Eleni Papadopoulou, Harrie Besselink, Kleopatra Mathianaki, Polyxeni Karakosta, Ariana Spanaki, Antonis Koutis, Leda Chatzi, Martine Vrijheid, Manolis Kogevinas

**Affiliations:** 1 Centre for Research in Environmental Epidemiology (CREAL), Barcelona, Spain; 2 Municipal Institute of Medical Research (IMIM-Hospital del Mar), Barcelona, Spain; 3 CIBER Epidemiología y Salud Pública (CIBERESP), Spain; 4 Pompeu Fabra University, Barcelona, Spain; 5 Parc de Salut Mar, Obstetrics and Gynecology Department, Barcelona, Spain; 6 National School of Public Health, Athens, Greece; 7 Biodetection Systems B.V., Amsterdam, the Netherlands; 8 Department of Social Medicine, Medical School, University of Crete, Heraklion, Crete, Greece; 9 Venizeleio Hospital, Heraklion, Crete, Greece

**Keywords:** anogenital distance, dioxin-like compounds, dioxins, DR CALUX®, persistent organic pollutants

## Abstract

Background: Anogenital distance in animals is used as a measure of fetal androgen action. Prenatal exposure to dioxins and dioxin-like compounds in rodents causes reproductive changes in male offspring and decreases anogenital distance.

Objective: We assessed whether *in utero* exposure to dioxins and dioxin-like compounds adversely influences anogenital distance in newborns and young children (median age, 16 months; range, 1–31 months).

Methods: We measured anogenital distance among participants of the “Rhea” mother–child cohort study in Crete and the Hospital del Mar (HMAR) cohort in Barcelona. Anogenital distance (AGD; anus to upper penis), anoscrotal distance (ASD; anus to scrotum), and penis width (PW) were measured in 119 newborn and 239 young boys; anoclitoral (ACD; anus to clitoris) and anofourchetal distance (AFD; anus to fourchette) were measured in 118 newborn and 223 young girls. We estimated plasma dioxin-like activity in maternal blood samples collected at delivery with the Dioxin-Responsive Chemically Activated LUciferase eXpression (DR CALUX®) bioassay.

Results: Anogenital distances were sexually dimorphic, being longer in males than females. Plasma dioxin-like activity was negatively associated with AGD in male newborns. The estimated change in AGD per 10 pg CALUX®–toxic equivalent/g lipid increase was –0.44 mm (95% CI: –0.80, –0.08) after adjusting for confounders. Negative but smaller and nonsignificant associations were observed for AGD in young boys. No associations were found in girls.

Conclusions: Male infants may be susceptible to endocrine-disrupting effects of dioxins. Our findings are consistent with the experimental animal evidence used by the Food and Agriculture Organization/World Health Organization to set recommendations for human dioxin intake.

Polychlorinated dibenzodioxins (PCDDs), dibenzofurans (PCDFs), and biphenyls (PCBs) constitute a group of widespread and persistent organic pollutants with 2,3,7,8-tetrachlorodibenzo-*p*-dioxin (TCDD, dioxin) being the most toxic member of this group (Birnbaum 1994b, 1995a, 1995b). PCDDs, PCDFs, and dioxin-like PCBs have long half-lives and therefore accumulate in the body. Food is the main source of dioxin exposure for humans, estimated at > 95% of the total intake for non-occupationally exposed persons (Parzefall 2002). Infant exposure starts *in utero*, through the placenta, and continues postnatally through breastfeeding.

The toxicity of PCDDs, PCDFs, and dioxin-like PCBs is traced mostly to their binding to the aryl hydrocarbon receptor (AhR). The AhR, on exposure to TCDD, translocates into the nucleus, where it heterodimerizes with the AhR nuclear translocator (ARNT). This complex then binds to its specific DNA recognition sites to activate the transcription of dioxin responsive genes (Hankinson 1995). The AhR induces expression of direct target genes such as the drug metabolizing enzyme genes *CYP1A1* and *CYP1A2* (Safe and Krishnan 1995). Furthermore, the ligand-activated AhR associates with estrogen or androgen receptors (ERα or AR) to regulate transcription as a functional unit (Ohtake et al. 2003, 2008). Although early studies focused on the AhR as mediating the biochemical response to xenobiotics, recent studies have revealed that, triggered by natural and endogenous ligands, AhR plays key endogenous regulatory roles in normal physiology and development (Abel and Haarmann-Stemmann 2010; Denison et al. 2011).

Because TCDD and other dioxin-like compounds exist as complex mixtures of various congeners throughout the environment, calculating total TCDD toxic equivalent (TEQ) concentration is the most relevant exposure measure in studies of health effects of dioxins and dioxin-like compounds (Warner et al. 2005). The Dioxin-Responsive Chemically Activated LUciferase eXpression (DR CALUX®) assay measures the ability of a chemical mixture to activate AhR-dependent gene expression of the firefly luciferase gene in genetically modified cell lines, and only small amounts of blood plasma are required for these measurements (Brouwer et al. 2004).

Anogenital distance (AGD), the distance from the anus to the genitalia, is a sensitive marker used by reproductive toxicologists in animal experiments as a measure of fetal androgen action. In rodents, perineal growth is dihydrotestosterone-dependent, males have a greater AGD than females, and use of AGD to sex newborns is common (Greenham and Greenham 1977; Marty et al. 2003). AGD usually tracks through life, varies by dose of antiandrogen, and can be predictive of other androgen-responsive outcomes such as hypospadias and cryptorchidism (Gray et al. 1999). Animal studies reviewed by the Joint FAO/WHO (Food and Agriculture Organization/World Health Organization) Expert Committee on Food Additives showed reduction in AGD as well as feminized sexual behavior in male offspring associated with TCDD exposure (FAO/WHO 2002).

In human studies AGD has been examined only in recent years (Huang et al. 2009; Longnecker et al. 2007; Miao et al. 2011; Ozkan et al. 2011; Romano-Riquer et al. 2007; Salazar-Martinez et al. 2004; Sathyanarayana et al. 2010; Suzuki et al. 2011; Swan et al. 2005; Thankamony et al. 2009; Torres-Sanchez et al. 2008). No human studies have reported on the relationship between *in utero* dioxin exposure and AGD in offspring, whereas AGD has been inversely associated with prenatal exposure to other environmental endocrine disruptors, namely phthalates, dichlorodiphenyldichloroethylene (DDE), and bisphenol A (BPA) (Miao et al. 2011; Swan et al. 2005; Torres-Sanchez et al. 2008).

In the present study of two mother–child cohorts in Greece and Spain, the DR CALUX® bioassay was used to measure dioxin-like activity in maternal plasma. We hypothesized that *in utero* exposure to dioxins would decrease AGD in newborns and children.

## Materials and Methods

*Study population*. The present study was based on data from the “Rhea” mother–child cohort study in Crete, Greece, and the Hospital del Mar (HMAR) cohort in Barcelona, Spain. Both studies are part of the Newborns and Genotoxic exposure risks (NewGeneris) project.

The Rhea study prospectively examines a population-based sample of pregnant women and their children at the prefecture of Heraklion, Crete, Greece. Methods are described in detail elsewhere (Chatzi et al. 2009). Women were identified from February 2007 through February 2008 at the time of the first ultrasound examination at the 10th–13th week of gestation, were residents in the study area, were > 16 years of age, and had no communication handicap. Face-to-face structured interviews along with self-administered questionnaires and medical records were used to obtain information on nutrition, occupational, and environmental exposures and lifestyle, socioeconomic, and psychological factors during pregnancy and birth.

A total of 1,765 eligible women were approached during the enrollment period, 1,610 (91%) agreed to participate, and 1,317 (82%) were followed up until delivery. Seven hundred blood samples, provided by the study participants at delivery, were analyzed for dioxin-like activity. Anthropometric measurements were conducted for 165 newborns (84 boys and 81 girls) and 732 young children (374 boys and 358 girls). The present analysis included 121 newborns (62 boys and 59 girls) and 462 young children (median age, 16.0 months, range, 1–31 months; 239 boys and 223 girls), all of whom were singletons with information on anthropometry and *in utero* dioxin-like activity measurements.

In the HMAR study women were informed by their midwife at the delivery room about the NewGeneris project. The inclusion criteria were age ≥ 18 years, singleton pregnancy, HIV and hepatitis B/C negative, non-excessive postpartum hemorrhage, and non-urgent cesarean section. A similar questionnaire to that of the Rhea study was administered to the mothers within the first 48 hr after delivery by a trained nurse. We analyzed 205 blood samples for dioxin-like activity, and conducted anthropometric measurements for 187 newborns (95 boys and 92 girls). One hundred sixteen newborns (57 boys and 59 girls) with information on anthropometry and *in utero* dioxin-like activity were included in this analysis.

All procedures involving human subjects were approved by the ethical committee of the University Hospital in Heraklion, Crete, and by the Clinical Research Ethical Committee at Hospital del Mar. All study participants provided written informed consent for themselves and their children.

*Physical examination*. Examiners of both cohorts received a common and extensive training before conducting the measurements. All AGD measurements were performed using a standardized analytical protocol based on the protocol used in a previous study (Swan SH, personal communication; Swan et al. 2005), which was modified to include additional measurements (Callegari et al. 1987; Salazar-Martinez et al. 2004). Minor changes, mostly regarding the child’s position during measurement, were made to adapt the protocol for young children.

In male participants we recorded AGD, the distance from the anterior base of the penis to the center of the anus; anoscrotal distance (ASD), the distance from the posterior base of the scrotum to the center of the anus; and penis width (PW), the diameter of the penis in its base. In girls we recorded, anoclitoral distance (ACD), the distance between the clitoris and the center of the anus; and anofourchettal distance (AFD), the distance from the posterior convergence of the fourchette to the center of the anus. Each measurement was repeated three times and the average of the three measurements was recorded. Weight, length, and head circumference were measured twice, and average values were used for analysis.

AGDs were measured with a Vernier digital calliper in increments of 0.01 mm (Cal C/PROOF 150MM IP67; TESA Technology, Renens, Switzerland). An electronic scale readable to increments of 0.001 kg was used to measure weight (model 354; Seca Corporation, Hamburg, Germany), a measuring mat was used to measure length (model 210; Seca Corporation), and a non-stretchable measuring tape was used to measure head circumference.

In the Rhea study three examiners conducted the measurements of newborns at the clinics and four examiners the measurements of young children at their homes. In the HMAR cohort, all measurements were conducted by a single examiner within the first 48 hr after delivery.

*Blood sample collection*. Maternal peripheral blood samples were collected in heparinized tubes (BD Vacutainer, Plymouth, UK) immediately after the delivery. The blood was centrifuged and the plasma was stored at –80°C until shipment to the Netherlands on dry ice.

*DR CALUX® bioassay*. Dioxin-like activity in maternal plasma samples was determined through the DR CALUX® assay at Biodetection Systems B.V., Amsterdam (www.bds.nl). The CALUX® assay is based on a genetically modified H4IIE rat hepatoma cell line, which contains the firefly luciferase reporter gene under the transcriptional control of AhR. When cells are exposed to dioxins or dioxin-like chemicals, through binding to the AhR, they express luciferase as well as proteins and enzymes associated with dioxin-responsive elements. With addition of the substrate luciferine for the luciferase enzyme, light is emitted. The amount of light emitted is proportional to the strength of the AhR binding. The luminance is calibrated with respect to 2,3,7,8-TCDD in units of toxic equivalency quantity (TEQs), and results are expressed as picograms CALUX®–TEQ/gram lipid. The DR CALUX® bioassay has previously been validated and used in human biomonitoring studies (Brouwer et al. 2004; Halldorsson et al. 2009; Koppen et al. 2001, 2009; Pauwels et al. 2000; Pedersen et al. 2010; Porpora et al. 2009; Van Den Heuvel et al. 2002). The protocol for sample processing has been presented elsewhere (Murk et al. 1997) and is described in detail in Supplemental Material, p. 2 (http://dx.doi.org/10.1289/ehp.1205221).

*Statistical analysis*. We used linear regression models to explore the associations between dioxin-like activity in maternal plasma and anogenital parameters. Samples below the limit of detection (LOD) were assigned a value equal to 0.5 × LOD before analyses for associations. Body dimensions have been found to be major predictors of AGD. All models included birth weight and weight at the time of examination for newborns and children respectively. We did not adjust for length because it was not a significant predictor of any of the outcomes in our study (*p* > 0.05). In addition to birth weight, each basic model for newborns included gestational age and cohort. In addition to weight at the time of examination, basic models for young children included age at examination and a variable indicating the examiner. We also ran fully adjusted models that included all potential confounders that predicted the outcome with *p* < 0.2 when added to the basic model for each age group.

In addition we modeled associations using weight-standardized *z*-scores of AGD as the outcome. In alternative analyses we adjusted for body size using weight percentiles for age based on WHO tables (WHO 2006). Generalized additive models (GAMs) were applied to explore the shape of the relationships between dioxin-like activity and AGDs and test departures from linearity. These models indicated linear relationships for all AGDs in newborns and young children. Analyses were conducted using STATA software, version 10.0 (StataCorp, College Station, TX, USA). The level of significance was set at *p* < 0.05 (two-sided).

## Results

*Participants’ characteristics*. Mothers of newborns and young children had a mean (± SD) age of 29.8 ± 5.4 years and 30.1 ± 4.7 years, respectively, and had a prepregnancy body mass index (BMI) within the normal range with median [interquartile range (IQR)] values of 23.4 (5.3) and 23.4 (5.1) kg/m^2^ respectively (Table 1). Most of the newborns (73.7%) and half (50.8%) of the young children were vaginally delivered, with a mean (± SD) birth weight of 3,277 ± 429.2 g and 3,167 ± 441.6 g respectively. Participants were mainly white European, multiparous nonsmokers living in urban areas. Percentages of boys and girls were similar in newborns (50.2% boys and 49.8% girls) and young children (51.7% boys and 48.3% girls).

**Table 1:** Maternal and child characteristics in newborns (*n* = 237) and young children (*n* = 462).^a^

Newborns			Young children		
Characteristic	n	Value	n	Value
Maternal characteristics
Country of residence (%)
Greece	121	49.0	456	100.0
Spain	116	51.0	0	0
Maternal age [years (mean ± SD)]	237	29.8 ± 5.4	456	30.1 ± 4.7
Missing	6
Prepregnancy BMI, kg/cm2 [median (IQR)]	224	23.4 (5.3)	456	23.4 (5.1)
Missing	13	6
Weight gain during pregnancy, kg [median (IQR)]	NAb	378	13.0 (7.0)
Missing	84
Maternal ethnicity (%)
Nonwhite European	59	25	0	0
White European	177	75	462	100
Missing	1
Parity (%)
Primiparous	95	42.0	155	34.7
Multiparous	131	58.0	292	65.3
Missing	11	15
Residence (%)
Urban	201	87.0	335	80.7
Rural	30	13.0	80	19.3
Missing	6	47
Maternal education (%)
Low	67	28.8	89	19.6
Medium	100	42.9	223	49.0
High	66	28.3	143	31.4
Missing	4	7
Delivery hospital (%)
Private	52	21.9	175	38.1
Public	185	78.1	284	61.9
Missing	3
Smoking during pregnancy (%)
No	154	66.7	358	78.5
Yes	77	33.3	98	21.5
Missing	6	6
Type of delivery (%)
Vaginal delivery	174	73.7	232	50.8
Cesarean section	62	26.3	225	49.2
Missing	1	5
Child characteristics
Sex (%)
Males	119	50.2	239	51.7
Females	118	49.8	223	48.3
Birth weight [g (mean ± SD)]	237	3,277 ± 429.2	454	3,167 ± 441.6
Missing	8
Birth length, cm [median (IQR)]	234	50.0 (2.0)	447	50.0 (3.0)
Missing	3	15
Gestational age, weeks [median (IQR)]	237	39.0 (2.0)	449	38.0 (1.0)
Missing	13
Weight at examination [g (mean ± SD)]	236	3,229 ± 446.7	456	11,224 ± 2066.3
Missing	1	6
Length at examination, cm [median (IQR)]	236	50.0 (2.0)	457	82.5 (11.0)
Missing	1	5
Age at examination, months [median (IQR)]	462	16.0 (11)
Head circumference at examination, cm [median (IQR)]	236	34.5 (1.8)	462	47.5 (3.0)
Missing	1
Breastfeeding (%)
Never	64	14.3
Ever	384	85.7
Missing	14
aMedian age, 16 months; range, 1–31 months. bData not available (NA) for the HMAR cohort.								

*Dioxin-like compounds and anogenital parameters*. Mean (± SD) AGDs were longer in male newborns (AGD = 48.8 ± 5.1 mm, ASD = 25.5 ± 4.8 mm) than in female (ACD = 35.0 ± 3.3 mm, AFD = 14.3 ± 3.0 mm) (Table 2). Similarly, in young children mean AGDs were longer in males (AGD = 80.7 ± 7.3 mm, ASD = 39.9 ± 6.9 mm) than females (ACD = 49.1 ± 6.0 mm, AFD = 21.7 ± 3.9 mm). The mean of PW was 10.7 ± 1.1 mm in newborns and 14.0 ± 1.7 mm in young boys. The mean of the samples was 52.3 ± 20.7 pg CALUX®–TEQ/g lipid in mothers of newborns and 49.7 ± 26.7 pg CALUX®–TEQ/g lipid in mothers of young children.

**Table 2:** Distribution of dioxin-like compounds in maternal plasma, AGDs, and penis width in newborns and young children.

Variables	Newborns (n = 237)									Young children (n = 462)^a^								
	n	Mean ± SD	Percentile					n	Mean ± SD	Percentile				
			25th	50th	75th	25th	50th			75th
Anogenital distances																				
Males																				
AGD (mm)	119	48.8 ± 5.1	45.5	48.2	51.9	237	80.7 ± 7.3	75.2	80.7	86.5
ASD (mm)	119	25.5 ± 4.8	22.4	25.2	28.8	239	39.9 ± 6.9	34.3	39.8	44.9
PW (mm)	117	10.7 ± 1.1	10.0	10.6	11.3	235	14.0 ± 1.7	12.8	14.1	14.9
Females
ACD (mm)	118	35.0 ± 3.3	32.7	34.8	37.1	223	49.1 ± 6.0	45.4	48.6	53.3
AFD (mm)	118	14.3 ± 3.0	12.4	14.2	15.7	223	21.7 ± 3.9	18.5	21.5	24.1
Dioxin-like compounds in maternal plasma																				
pg TEQ/g lipid	237	52.3 ± 20.7	42.5	53.6	66.0	462	49.7 ± 26.7	34.7	50.3	63.5
Percent < LOD	7.6	10.6
aMedian age, 16 months; range, 1–31 months.																				

Mean weight at examination of newborns was lower among newborn male and female children whose mothers had CALUX®–TEQs above the median (> 53.6 pg CALUX®–TEQ/g lipid) compared with children whose mothers had values below the median, although differences were small and not statistically significant (Table 3). In young children, weight at examination was higher in children whose mothers had dioxin-like activity above the median (> 50.3 pg CALUX®–TEQ/g lipid), with a significant difference in young males. Compared with newborn children whose mothers had low dioxin-like activity, newborn children of mothers with high activity had small nonsignificant decreases in AGD in males (48.4 mm vs. 49.1 mm, *p* = 0.617) and ACD and AFD in females (34.9 mm vs. 35.1 mm, *p* = 0.592 and 14.1 mm vs. 14.4 mm, *p* = 0.892 respectively).

**Table 3:** Mean (± SD) of physiological variables in newborns (*n* = 237) and young boys (*n* = 462) categorized by median levels of dioxin-like activity in maternal plasma expressed in pg CALUX®–TEQ/g lipid.

pg CALUX^®^–TEQ/g lipid^a,b^								
n	Low	n	High	p-Value^c^
Newborns
Males
Gestational age (weeks)	62	38.8 ± 1.7	57	38.7 ± 1.4	0.374
Weight at examination (g)	61	3,305 ± 441.6	57	3,282 ± 386.9	0.887
AGD (mm)	62	49.1 ± 5.3	57	48.4 ± 4.9	0.617
ASD (mm)	62	25.3 ± 5.3	57	25.7 ± 4.2	0.374
PW (mm)	62	10.6 ± 1.1	55	10.9 ± 1.0	0.163
Females
Gestational age (weeks)	57	38.8 ± 1.3	61	38.5 ± 1.8	0.338
Weight at examination (g)	57	3,190 ± 506.0	61	3,140 ± 469.7	0.615
ACD (mm)	57	35.1 ± 3.8	61	34.9 ± 2.8	0.592
AFD (mm)	57	14.4 ± 3.1	61	14.1 ± 2.6	0.892
Young children
Males
Weight at examination (g)	108	11,239 ± 2128.8	126	11,843 ± 1967.0	0.048*
Age at examination (months)	110	17.2 ± 7.2	129	18.5 ± 5.7	0.207
AGD (mm)	110	80.0 ± 7.8	127	81.2 ± 6.9	0.142
ASD (mm)	110	39.6 ± 7.1	129	40.2 ± 6.7	0.471
PW (mm)	110	14.0 ± 1.7	125	13.9 ± 1.7	0.914
Females
Weight at examination (g)	120	10,763 ± 2175.6	102	10,984 ± 1810.9	0.645
Age at examination (months)	121	16.9 ± 7.3	102	18.2 ± 6.3	0.162
ACD (mm)	121	48.7 ± 6.1	102	49.5 ± 6.0	0.460
AFD (mm)	121	21.8 ± 3.8	102	21.6 ± 4.0	0.760
aMedian levels of plasma dioxin-like compounds in the low-level and high-level newborn groups were 42.5 (IQR = 21.2; range, 6–53.6) and 66.1 (IQR = 12.5; range, 53.7–106.3) pg CALUX®–TEQ/g lipid, respectively; median levels of plasma dioxin-like compounds in the low-level and high-level young children groups were 34.7 (IQR = 22.0; range, 6–50.2) and 63.5 (IQR = 14.4; range, 50.4–225.7) pg CALUX®–TEQ/g lipid, respectively. bMedian value of dioxin-like compounds in the newborn group was 53.6pg CALUX®–TEQ/g lipid; median value of dioxin-like compounds in the young children group was 50.3 pg CALUX®–TEQ/g lipid. Values above median were categorized as high whereas values below median were categorized as low in both age groups. cKruskal–Wallis test. *p < 0.05.										

*Relationship between plasma dioxin-like activity and anogenital parameters*. Plasma dioxin-like activity was negatively associated with AGD in male newborns (Table 4). The estimated change in newborn AGD per 10 pg CALUX®–TEQ/g lipid was –0.41 mm (95% CI: –0.77, –0.06) according to the basic model (adjusted for birth weight, gestational age and cohort), with a similar estimate based on the fully-adjusted model (Table 4). Analyses by country also were similar (–0.43 mm; 95% CI: –0.88, 0.02 for Rhea and –0.39 mm; 95% CI: –1.02, 0.25 for HMAR in fully-adjusted models). Negative but not statistically significant associations were observed for ASD in male newborns (–0.14 mm; 95% CI: –0.51, 0.23 and –0.25 mm; 95% CI: –0.61, 0.11 for the basic and fully adjusted models, respectively). Small nonsignificant negative associations were observed for AGD and the weight standardized *z*-score of AGD in young boys. All associations were close to the null for girls, except for small positive but nonsignificant associations with ACD in young girls [see Supplemental material, Table S1 (http://dx.doi.org/10.1289/ehp.1205221)]. All estimates were similar to those reported when models were adjusted for weight percentile according to age instead of weight (data not shown).

**Table 4:** Associations between a 10-pg increase in maternal DR CALUX®–TEQ/g lipid and anogenital distances and penis width in newborn and young boys [β (95% CI)].

Outcomes	Change per 10-pg increase in DR CALUX^®^–TEQ/g lipid						
	n	Basic model^a^		Fully adjusted model	
Newborns
AGD (mm)	115	–0.41	(–0.77, –0.06)	–0.44b	(–0.80, –0.08)
ASD (mm)	112	–0.14	(–0.51, 0.23)	–0.25c	(–0.61, 0.11)
PW (mm)	116	0.03	(–0.05, 0.11)	0.02d	(–0.06, 0.09)
Young boys
AGD (mm)	207	–0.13	(–0.44, 0.18)	–0.07e	(–0.39, 0.24)
ASD (mm)	218	0.06	(–0.25, 0.38)	0.08f	(–0.23, 0.39)
PW (mm)	215	–0.05	(–0.11, 0.02)	–0.04g	(–0.10, 0.03)
Weight standardized z-scores of anogenital distancesh								
AGD z-score	207	–0.01	(–0.06, 0.03)	–0.01	(–0.05, 0.04)
ASD z-score	218	0.01	(–0.03, 0.06)	0.01	(–0.03, 0.06)
PW z-score	215	–0.03	(–0.07, 0.01)	–0.02	(–0.07, 0.02)
aBasic model adjusted for birth weight, gestational age and cohort in newborns, and for weight and age at examination and examiner in young boys. bBasic model plus maternal ethnicity and maternal education. cBasic model plus maternal ethnicity, smoking during pregnancy, and type of delivery. dBasic model plus maternal age and delivery hospital. eBasic model plus delivery hospital, maternal education, smoking during pregnancy, and residence. fBasic model plus maternal age, parity, prepregnancy BMI, and maternal education. gBasic model plus maternal age, parity, delivery hospital, and maternal education. hAll models for weight standardized z-scores of anogenital distances are adjusted for the same variables as in the models for the crude measurements of anogenital distances in young boys without weight at the time of measurement.								

**Figure 1:**
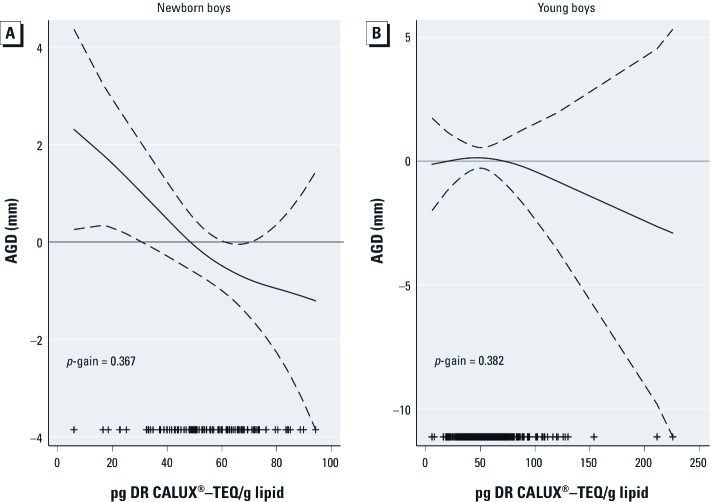
GAMS; adjusted associations (95% CIs) between dioxin-like activity and AGD in newborn (*A*) and young (*B*) boys. (*A*) Adjusted for birth weight, gestational age, cohort, maternal ethnicity, and maternal education. (*B*) Adjusted for weight and age at examination, examiner, delivery hospital, maternal education, smoking during pregnancy, and residence. ++, observations.

GAMs examining the shape of the relationships between dioxin-like activity in maternal plasma expressed in picograms CALUX®–TEQ per gram lipid and AGD (millimeters) (Figure 1) showed no significant departures from linearity, both for newborn (*p*-gain = 0.367) and young boys (*p*-gain = 0.382).

## Discussion

In the present study of two population-based mother–child cohorts in Greece and Spain, prenatal exposure to dioxins and dioxin-like compounds was negatively associated with AGD in males in the context of overall low-level exposures in the general population. We found no evidence that exposure was related to reduced AGDs in girls. Our results are consistent with animal studies as reviewed by the Joint FAO/WHO Expert Committee on Food Additives that noted that the most sensitive adverse effects were on the development of male offspring of rats after prenatal exposure to TCDD (FAO/WHO 2002).

To our knowledge, this is the first study to estimate the effect of dioxins on the development of the human genital system. Our findings are supported by several animal studies where prenatal and lactational exposure to TCDD was associated with a reduced AGD (Gray et al. 1995; Jin et al. 2008, 2010; Ohsako et al. 2001, 2002). Mocarelli et al. (2011) showed that semen quality and sperm counts were reduced in young men with *in utero* and lactational exposure to dioxin in the Seveso accident.

Although the health effect of *in utero* exposure to dioxins on the development of the human reproductive organs is largely unknown, the effect of other endocrine disruptors has been explored. Two studies have found that *in utero* exposure to phthalates was associated with shortened AGD (Suzuki et al. 2011; Swan et al. 2005). However, a third study reported no statistical association between phthalates and male newborns’ AGI (AGD/weight) (Huang et al. 2009). No effect of prenatal exposure to DDE on AGD at birth was reported by two studies in Mexico (Longnecker et al. 2007; Torres-Sanchez et al. 2008) although the smaller study reported a significant reduction in one of the indices measured (Anal Position Index) which is a non-age-dependent measurement of AGD (Torres-Sanchez et al. 2008). A recent study reported that *in utero* exposure to BPA was associated with decreased AGD (Miao et al. 2011).

It has been suggested that human hypospadias and cryptorchidism may be associated with reduced AGDs as a result of endocrine disruption (Hsieh et al. 2008). Moreover, findings of recent studies have linked shorter AGD to reproductive parameters in adulthood. Decreased AGD predicted poorer semen quality (Mendiola et al. 2011), and men who had fathered a child had a longer AGD than infertile urology clinic patients (Eisenberg et al. 2011). Men with hypogonadal testosterone levels (< 300 ng/dL) had a significantly shorter AGD compared with men with higher testosterone levels (Eisenberg et al. 2012). In children of the Rhea cohort, neonatal AGD predicted the corresponding genitalia measure at early childhood (Papadopoulou et al. 2013).

In the present study we measured two genital distances, AGD and ASD, but found significant associations with dioxin exposure only for AGD, as did Swan et al. (2005) with exposure to phthalates. On the other hand Mendiola et al. (2011) saw significant associations with sperm parameters only for ASD. These findings suggest that different genitalia measurements may reflect androgen exposures at different stages of life.

We found no evidence that *in utero* exposure to dioxins and dioxin-like compounds is associated with female AGDs. Of the two other studies that examined AGD in females, one reported no associations with prenatal DDE exposure (Torres-Sanchez et al. 2008) and the other found prenatal phthalates exposure to be associated with shorter AGI (Huang et al. 2009). Animal studies suggest that some effects of environmental chemicals, including TCDD, may not be detected until puberty or even later in life (Birnbaum 1994a; Gray and Ostby 1995; Heimler et al. 1998; Wolf et al. 1999). In humans there is some evidence that higher exposure to dioxins and dioxin-like compounds is associated with delayed breast development (Den Hond et al. 2002; Leijs et al. 2008). Hence, further follow-up of the girls in our study is needed to evaluate possible effects of dioxins on their reproductive health.

Our results provided some evidence of an adverse effect of dioxins on AGDs of young boys, although estimated effects were small and not statistically significant. The few epidemiological studies which have explored the relationship between AGDs and prenatal exposures have collected their measurements at birth, and only Swan et al. (2005) explored phthalate exposure in relation to AGD in a study of young boys 2–36 months of age. Child’s body size is positively associated with AGD (Ozkan et al. 2011; Romano-Riquer et al. 2007; Salazar-Martinez et al. 2004; Sathyanarayana et al. 2010; Thankamony et al. 2009), so a possible reduction due to prenatal exposure might be masked by growth during the first years of life. Moreover, AGDs in childhood could also be affected by early life exposures. Breastfeeding is the main source of exposure to dioxins in early life, but in our study population the duration of breastfeeding was short (median, 2 months) and not associated with AGD (data not shown). Although it would have been ideal to measure the same children at birth and in early childhood, this was not possible due to the designs of the two studies.

In this study, exposure to dioxins and dioxin-like compounds was estimated with the DR CALUX® bioassay. Methods for quantification of dioxin exposure include sensitive and specific techniques such as high-resolution gas chromatography/mass spectrometry. However, these methods are time-consuming and expensive and require large sample volumes (Warner et al. 2005). Although the DR CALUX® does not quantify specific compounds, it provides an overall biological response/potency of mixture that will reflect the effects of possible interactions (synergistic, additive and/or antagonistic interaction) between congeners (Long et al. 2007).

Mean plasma levels of our study (52.3 ± 20.7 and 49.7 ± 26.7 pg CALUX®–TEQ/g lipid in newborns and young children, respectively) were similar to those in other published European studies, except for a Dutch study (mean, 103.7 pg TEQ/g lipid) conducted in the early 1990s (Koopman-Esseboom et al. 1994). Mean levels in two studies of pregnant women in Denmark were 46.0 and 37.0 pg CALUX®–TEQ/g lipid respectively (Halldorsson et al. 2009; Pedersen et al. 2010). A case–control study on endometriosis in Rome reported 18.6 and 20.0 pg TEQ/g lipid in cases and controls (Porpora et al. 2009). Moreover, Belgian studies in young and middle aged women, adolescents, and newborns have reported 46.8 (Pauwels et al. 2000), 36.0 (Koppen et al. 2001), 28.6 and 34.9 (in girls and boys respectively) (Van Den Heuvel et al. 2002), and 23.0 pg CALUX®–TEQ/g lipid (Koppen et al. 2009).

## Conclusions

Our results suggest that male infants may be susceptible to endocrine-disrupting effects of dioxins even in the context of overall low-level exposure in the general population. Our findings are consistent with the experimental animal evidence used by FAO/WHO to set recommendations for human dioxin intake.

## Supplemental Material

(225 KB) PDF
