# Supplementary material for: In Utero Exposure to Dioxins and Dioxin-like Compounds and Anogenital Distance in Newborns and Infants
Source: Environ Health Perspect. 2012 Nov 19;121(1):125–30. doi: 10.1289/ehp.1205221 (PMC3553434; doi:10.1289/ehp.1205221)
Supplement: (225 KB) PDF [file ehp.1205221.s001.pdf]

Supplemental Material

Title: *In Utero* Exposure to Dioxins and Dioxin-like Compounds and Anogenital Distance in Newborns and Infants.

Marina Vafeiadi[1,2,3,4] Silvia Agramunt[1,2,5], Eleni Papadopoulou[1,2,3,4,6], Harrie Besselink[7], Kleopatra Mathianaki[8], Polyxeni Karakosta[8], Ariana Spanaki[9], Antonis Koutis[8], Leda Chatzi[8], Martine Vrijheid[1,2,3], Manolis Kogevinas[1,2,3,6]

[1]Centre for Research in Environmental Epidemiology (CREAL), Barcelona, Spain

[2]Municipal Institute of Medical Research (IMIM-Hospital del Mar), Barcelona, Spain

[3]CIBER Epidemiología y Salud Pública (CIBERESP), Spain

[4]Pompeu Fabra University, Barcelona, Spain

[5]Parc de Salut Mar, Obstetrics and Gynecology Department, Barcelona, Spain

[6]National School of Public Health, Athens, Greece

[7]Biodetection Systems B.V., Amsterdam, The Netherlands

[8]Department of Social Medicine, Medical School, University of Crete, Heraklion, Greece

[9]Venizeleio Hospital, Heraklion, Crete, Greece

Materials and Methods

Protocol for the DR CALUX bioassay

The total fat from approximately 1mL of plasma was extracted in 97% hexane 3% diethyl ether solution by shake-solvent extraction. The extracted fat was then passed through two acid silica columns topped with sodium sulfate (first 20% and then 30% H_2SO_4) to remove matrix components. The purified extracts were evaporated under nitrogen and re-dissolved in 8µL of dimethyl sulfoxide (DMSO). The CALUX® cells were cultured in α-MEM culture medium supplemented with 10% (v/v) FCS under standard conditions (37°C, 5% CO_2, 100% humidity) and were exposed in triplicate to cleaned extracts for 24 hours in 96-well microtiter plates. After incubation, the cells were lysed. A luciferine containing solution was added and the luciferase activity was measured using a luminometer. Each 96-well microtiter plate contained a 2,3,7,8-TCDD calibration range (0–3 pM 2,3,7,8-TCDD per well), a DMSO control, a procedure blank and an internal reference material. Total DR CALUX®-TEQ in the samples was determined by interpolation from the fitted 2,3,7,8-TCDD calibration curve and corrected for procedure blank. The limit of detection (LOD) was calculated as the signal measured from the DMSO control on each plate plus three times its standard deviation. Lipid content of the plasma was determined gravimetrically.

Results

Table S1. Associations between a 10 pg increase in maternal DR CALUX®-TEQ/g lipid and anogenital distances in newborn and young girls.

	Change per 10 pg increase in DR CALUX®-TEQ/g lipid				
		Basic model[a]		Fully-adjusted model	
Outcomes	N	β	95%CI	β	95%CI
Newborns					
ACD (mm)	117	0.04	-0.23, 0.30	0.07[b]	-0.20, 0.35
AFD (mm)	109	-0.004	-0.28, 0.28	-0.03[c]	-0.32, 0.26
Young girls					
ACD (mm)	219	0.10	-0.19, 0.39	0.13[d]	-0.16, 0.41
AFD (mm)	218	0.05	-0.13, 0.23	0.04[e]	-0.14, 0.22
*Weight standardized z-scores of anogenital distances**					
ACD z-score	219	0.02	-0.03, 0.07	0.02	-0.03, 0.07
AFD z-score	218	0.004	-0.04, 0.05	0.002	-0.05, 0.05

[a]Basic model adjusted for birth weight, gestational age and cohort in newborns and for weight and age at examination and examiner in young girls.
[b]Basic model plus maternal ethnicity, delivery hospital, maternal education and smoking during pregnancy
[c]Basic model plus pre pregnancy BMI and residence
[d]Basic model plus smoking during pregnancy and pre pregnancy BMI
[e]Basic model plus parity and pre pregnancy BMI

*All models for weight standardized z-scores of anogenital distances are adjusted for the same variables as in the models for the crude measurements of anogenital distances in young girls without weight at the time of measurement.
